# Development of a Symptom-Based Tool for Screening of Children at High Risk of Preschool Asthma

**DOI:** 10.1001/jamanetworkopen.2022.34714

**Published:** 2022-10-06

**Authors:** Myrtha E. Reyna, Ruixue Dai, Maxwell M. Tran, Vanessa Breton, Maria Medeleanu, Wendy Y. W. Lou, Rachel E. Foong, Melanie Emmerson, Christoffer Dharma, Kozeta Miliku, Diana L. Lefebvre, Elinor Simons, Meghan B. Azad, Moira Chan-Yeung, Allan B. Becker, Piush J. Mandhane, Stuart E. Turvey, Graham L. Hall, Theo J. Moraes, Malcolm R. Sears, Padmaja Subbarao

**Affiliations:** 1 Department of Pediatrics, The Hospital for Sick Children, Toronto, Ontario, Canada; 2 Dalla Lana School of Public Health, University of Toronto, Toronto, Ontario, Canada; 3 Wal-yan Respiratory Centre, Children's Lung Health, Telethon Kids Institute, Perth, Western Australia, Australia; 4 School of Allied Health, Curtin University, Perth, Western Australia, Australia; 5 Department of Precision Genomics, Intermountain Healthcare, Salt Lake City, Utah; 6 Department of Medicine, McMaster University, Hamilton, Ontario, Canada; 7 Department of Pediatrics and Child Health, University of Manitoba, Manitoba, Winnipeg, Canada; 8 Department of Medicine, University of British Columbia, Vancouver, British Columbia, Canada; 9 Department of Pediatrics, University of Alberta, Edmonton, Alberta, Canada; 10 Department of Pediatrics, BC Children’s Hospital, The University of British Columbia, Vancouver, British Columbia, Canada

## Abstract

**Importance:**

Despite advances in asthma therapeutics, the burden remains highest in preschool children; therefore, it is critical to identify primary care tools that distinguish preschool children at high risk for burdensome disease for further evaluation. Current asthma prediction tools, such as the modified Asthma Predictive Index (mAPI), require invasive tests, limiting their applicability in primary care and low-resource settings.

**Objective:**

To develop and evaluate the use of a symptom-based screening tool to detect children at high risk of asthma, persistent wheeze symptoms, and health care burden.

**Design, Setting, and Participants:**

The cohort for this diagnostic study included participants from the CHILD Study (n = 2511) from January 1, 2008, to December 31, 2012, the Raine Study from January 1, 1989, to December 31, 2012 (n = 2185), and the Canadian Asthma Primary Prevention Study (CAPPS) from January 1, 1989, to December 31, 1995 (n = 349), with active follow-up to date. Data analysis was performed from November 1, 2019, to May 31, 2022.

**Exposures:**

The CHILDhood Asthma Risk Tool (CHART) identified factors associated with asthma in patients at 3 years of age (timing and number of wheeze or cough episodes, use of asthma medications, and emergency department visits or hospitalizations for asthma or wheeze) to identify children with asthma or persistent symptoms at 5 years of age.

**Main Outcomes and Measures:**

Within the CHILD Study cohort, CHART was evaluated against specialist clinician diagnosis and the mAPI. External validation was performed in both a general population cohort (Raine Study [Australia]) and a high-risk cohort (CAPPS [Canada]). Predictive accuracy was measured by sensitivity, specificity, area under the receiver operating characteristic curve (AUROC), and positive and negative predicted values.

**Results:**

Among 2511 children (mean [SD] age at 3-year clinic visit, 3.08 [0.17] years; 1324 [52.7%] male; 1608 of 2476 [64.9%] White) with sufficient questionnaire data to apply CHART at 3 years of age, 2354 (93.7%) had available outcome data at 5 years of age. CHART applied in the CHILD Study at 3 years of age outperformed physician assessments and the mAPI in predicting persistent wheeze (AUROC, 0.94; 95% CI, 0.90-0.97), asthma diagnosis (AUROC, 0.73; 95% CI, 0.69-0.77), and health care use (emergency department visits or hospitalization for wheeze or asthma) (AUROC, 0.70; 95% CI, 0.61-0.78). CHART had a similar predictive performance for persistent wheeze in the Raine Study (N = 2185) in children at 5 years of age (AUROC, 0.82; 95% CI, 0.79-0.86) and CAPPS (N = 349) at 7 years of age (AUROC, 0.87; 95% CI, 0.80-0.94).

**Conclusions and Relevance:**

In this diagnostic study, CHART was able to identify children at high risk of asthma at as early as 3 years of age. CHART could be easily incorporated as a routine screening tool in primary care to identify children who need monitoring, timely symptom control, and introduction of preventive therapies.

## Key Points

**Question:**

Can a symptom-based screening tool (CHILDhood Asthma Risk Tool [CHART]) improve the ability to identify children at risk of persistent asthma symptoms and health care use?

**Findings:**

In this diagnostic study of 2354 children with validation in 2 external cohorts, CHART identified infants from the general population at high risk of asthma, persistent symptoms, and health care burden without the need for invasive and time-consuming tests.

**Meaning:**

These findings suggest that CHART may be incorporated as a routine screening tool in primary care settings to trigger timely treatment initiatives and promote active disease monitoring.

## Teaser

This diagnostic study of children in the general population examines whether the use of a symptom-based screening tool can identify children at high risk for asthma, persistent symptoms, and health care burden.

## Introduction

*Asthma* is an umbrella term used to describe a heterogeneous condition characterized by airflow limitation and variable wheeze symptoms.^1,2^ Asthma affects nearly 330 million individuals worldwide and carries a high economic burden, accounting for 1% to 2% of the health care budget of developed countries.^3^ In Canada, asthma is the leading cause of hospitalization among children, with the highest burden disproportionately shared in those younger than 5 years.^4^

Wheezing, a key defining trait of asthma,^5^ is extremely common in preschool children, being reported at least once in 30% to 50% of all children. However, the utility of wheezing in diagnosing childhood asthma is debated in part because of its variable course in early life, with nearly half of preschool children with wheeze experiencing a full remission by school age.^6^ Nonetheless, a widely held belief that preschool wheeze is a benign condition has been refuted in recent years because persistence of preschool wheezing symptoms to school age, even if associated with subsequent remission, is linked to lower lung function and chronic lung disease.^7^

Beyond a diagnostic label, the goal of standardized screening tools is timely identification of children at increased risk of symptom persistence and health care use. Tools such as the Asthma Predictive Index and its modified version (mAPI) have been implemented in practice with the goal of predicting school-age asthma.^6,8,9,10,11,12^ However, the applicability of these tools in primary care is limited given the need for invasive testing, such as blood or allergy skin prick tests, which are challenging in young children. Other tools, such as the Persistent Asthma Predictive Score, Predicting Asthma Risk in Children, or Pediatric Asthma Risk Score, either have been developed in children predisposed to asthma, require invasive tests, or are not validated in general populations.^6,8,9,10,13,14,15,16^ Hence, novel, pragmatic, and inexpensive screening tools that allow for the earlier identification of children at high risk of asthma are needed as a first step in busy primary practices. Timely detection of children requiring further evaluation for therapeutic intervention may aid in the prevention of asthma-related health care use and morbidity.

In this study, we leveraged data from the CHILD Study to develop a symptom-based screening tool to identify 3-year-old children at high risk of asthma, persistent wheeze symptoms, and health care burden at 5 years of age. Validation was performed in 2 external cohorts: the Raine Study in Australia and the Canadian Asthma Primary Prevention Study (CAPPS). To our knowledge, this is the first symptom-based preschool screening tool developed in a cohort of unselected individuals and validated in both general and high-risk cohorts.^13^

## Methods 

### Study Design

The CHILD Study recruited 3454 eligible women and their offspring between January 1, 2008, and December 31, 2012, across 4 Canadian sites (Toronto, Edmonton, Vancouver, and Manitoba). A total of 3224 women continued in the study until their offspring reached 5 years of age (eMethods and eAppendix in the Supplement).^17^ Parents answered child health questionnaires at multiple time points up to 5 years, and children attended clinic visits at 1, 3, and 5 years. Children with complete questionnaire and clinic data at 3 and 5 years were included in this analysis. Informed consent was explained verbally during clinic visits and provided in written form for parents or legal guardian signature. This diagnostic study was approved by the Hamilton Integrated Research Ethics Board, the universities of Manitoba, Alberta, and British Columbia, and The Hospital for Sick Children (Toronto, Ontario, Canada). This study followed the Transparent Reporting of a Multivariable Prediction Model for Individual Prognosis or Diagnosis (TRIPOD) reporting guideline.

### Development of CHART

**Figure 1.**
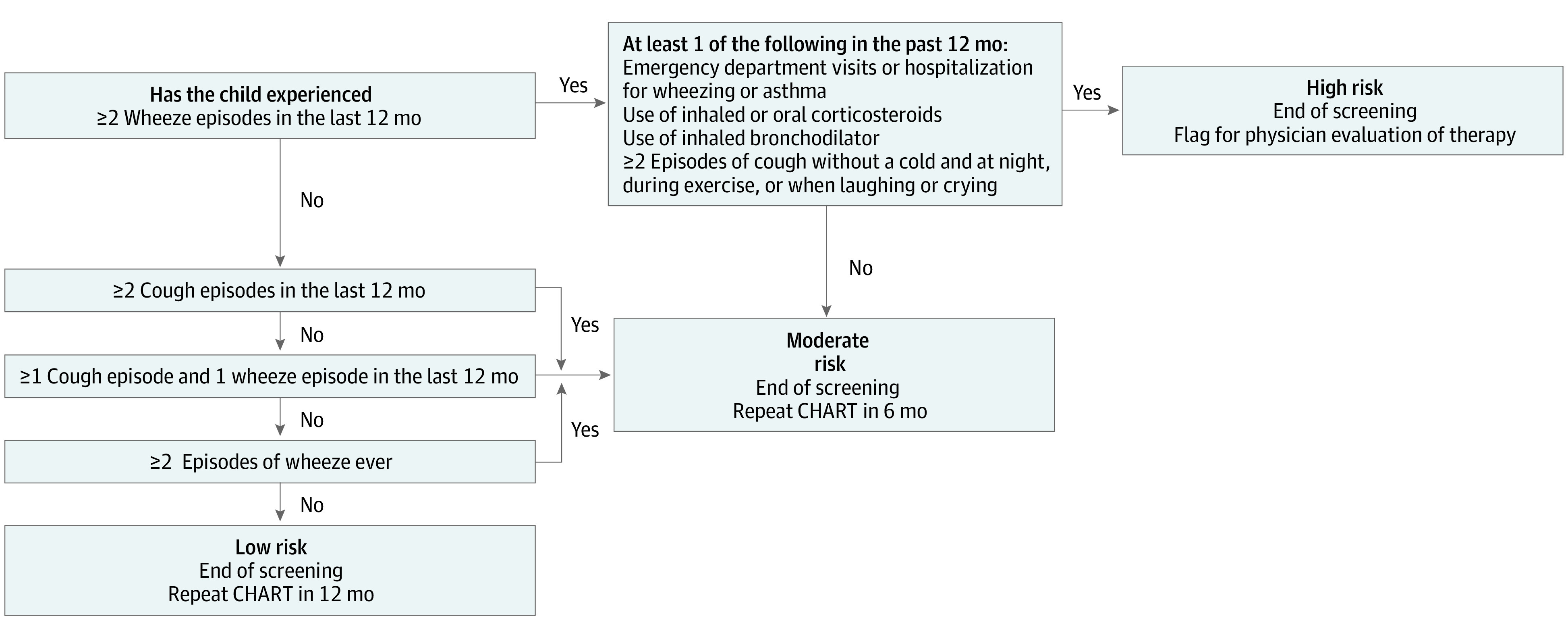
CHILDhood Asthma Risk Tool (CHART)

Children at risk of future asthma and persistent symptoms are categorized by the CHILDhood Asthma Risk Tool (CHART) as having high, moderate, or low risk based on symptoms reported before 3 years of age; follow-up actions are recommended for each group (Figure 1). CHART was developed following a structural logic approach in which combinations of asthma predictors before 3 years of age identified from published literature and clinical expertise were tested iteratively (eFigure 1 and eTable 1 in the Supplement). Predictors considered were timing and number of wheeze or cough episodes, use of inhaled corticosteroids, use of oral corticosteroids, use of inhaled bronchodilators, and emergency department (ED) visits and hospitalizations for asthma or wheeze. Children with 2 or more episodes of wheeze in the past year, concurrent with ED visits, hospitalizations, asthma medication, or frequent dry cough, are classified as being at high risk (eFigure 1 in the Supplement); children with only cough episodes or cough episodes plus 1 episode of wheeze occurring before the past 12 months are classified as being at low risk. Unlike stepwise methods, our approach of prespecification of predictors overcomes issues of overfitting and poor generalization to other data sets.^18^ To evaluate the importance of predictors obtained from invasive tests, a sensitivity analysis was performed, adding skin prick test (at 3 years of age) and blood eosinophil test (at 1 year of age) data into CHART.

### Study Outcomes

Within the CHILD cohort, 3-year diagnoses using CHART, mAPI, in-study physician diagnosis, and parent-reported external physician diagnosis were analyzed separately to predict persistent wheeze, asthma, and health care burden at 5 years of age. Predictive accuracy was additionally compared with that obtained from the published Pediatric Asthma Risk Score, which requires polysensitization, ethnicity, and parental history information.^16^

The outcome of persistent wheeze was defined as 2 or more wheeze episodes annually at both 3 and 5 years of age. In-study physician asthma diagnosis by a pediatric asthma specialist at 5 years was considered the criterion standard (eTable 2 in the Supplement). All in-study physicians were blinded to our screening tool before diagnosis. Health care burden at 5 years was defined as use of inhaled or oral corticosteroids or bronchodilators or having ED visits or hospitalizations for asthma and/or wheeze.

CHART performance was explored in 2 external cohorts: at 5 years of age in the Raine Study,^19,20^ a general-population Australian cohort, and at 7 years of age in the CAPPS cohort,^21,22^ a high-risk asthma Canadian cohort. The published asthma definitions within each study were used as the main point of comparison for CHART performance. Persistent wheeze and health care burden were defined as in the CHILD Study and were obtained from routine questionnaires in each of the external cohorts (description of the external cohorts and their variables are given in eTable 2 in the Supplement).

### Statistical Analysis

The ability of CHART, mAPI, in-study physician, and external physician diagnosis to predict persistent wheeze, asthma, and health care burden was measured by sensitivity, specificity, positive predictive value (PPV), negative predicted value (NPV), and area under the receiving operator curve (AUROC) obtained from univariate logistic regression.^18^ In all CHART diagnosis models, the high-risk group was compared with the moderate- and low-risk groups together. The main analysis in the CHILD Study included outcomes at 5 years and predictors at 3 years. Statistical significance was considered at the α = .05 level. All analyses were completed in SAS software, version 9.4 (SAS Institute Inc). Data analysis was performed from November 1, 2019, to May 31, 2022.

## Results

### Characteristics of the CHILD Study Sample

Among 2511 children (mean [SD] age at 3-year clinic visit, 3.08 [0.17] years; 1324 [52.7%] male and 1187 [47.3%] female) with sufficient questionnaire data to apply CHART at 3 years of age, 2354 (93.7%) had available outcome data at 5 years (eFigure 2 in the Supplement). The sample did not differ significantly from the overall CHILD population for most characteristics, but had lower smoke exposure compared with the full CHILD study (Table).

**Table.** Comparison of Study Sample at 3 and 5 Years of Age With Full CHILD Study Cohort^a^

Characteristic	Study sample at 3 y (n = 2511)	Study sample at 5 y (n = 2354)	Full CHILD Study cohort (n = 3454)
Sex			
Male	1324/2511 (52.7)	1250/2354 (53.1)	1816/3454 (52.6)
Female	1187/2511 (47.3)	1104/2354 (46.9)	1638/3454 (47.4)
Study center			
Edmonton	532/2511 (21.2)	485/2354 (20.6)	807/3454 (23.4)
Toronto	549/2511 (21.9)	503/2354 (21.4)	809/3454 (23.4)
Vancouver	645/2511 (25.7)	610/2354 (25.9)	783/3454 (22.7)
Manitoba	785/2511 (31.3)	756/2354 (32.1)	1055/3454 (30.5)
Race and ethnicity			
African American or Black	27/2476 (1.1)	25/2324 (1.1)	40/3355 (1.2)
East Asian	80/2476 (3.2)	76/2324 (3.3)	102/3355 (3.0)
South Asian	50/2476 (2.0)	45/2324 (2.0)	78/3355 (2.3)
Southeast Asian	62/2476 (2.5)	60/2324 (2.6)	81/3355 (2.4)
White	1608/2476 (64.9)	1514/2324 (65.1)	2162/3355 (64.4)
Mixed	598/2476 (24.2)	558/2324 (24.0)	804/3355 (24.0)
Other^b^	50/2476 (2.0)	44/2324 (1.9)	88/3355 (2.7)
Exact age at 3-y clinic visit, mean (SD), y	3.08 (0.17)	NA	3.08 (0.19)
Exact age at 5-y clinic visit, mean (SD), y	NA	5.08 (0.19)	5.10 (0.21)
Bronchodilator use^c^	247/2509 (9.8)	229/2352 (9.7)	274/2780 (9.9)
Corticosteroid use^c^	277/2509 (11.0)	263/2352 (11.2)	303/2781 (10.9)
Any wheeze^c^	414/2511 (16.5)	387/2354 (16.4)	489/2996 (16.3)
≥2 Wheeze episodes^c^	220/2511 (8.8)	206/2354 (8.8)	264/2996 (8.8)
ED visit or hospitalization for asthma or wheezing^c^	81/2321 (3.5)	76/2183 (3.5)	86/2555 (3.4)
Prenatal or postnatal smoke exposure up to 3 y	362/1843 (19.6)	328/1738 (18.9)	578/2214 (26.1)
Parent-reported external physician–diagnosed asthma at 3 y	112/2511 (4.5)	104/2354 (4.4)	114/2602 (4.4)
In-study physician diagnosis at 3 y			
Definite asthma	141/2511 (5.6)	132/2354 (5.6)	176/2889 (6.1)
Possible asthma	169/2511 (6.7)	158/2354 (6.7)	203/2889 (7.0)
No asthma	2201/2511 (87.7)	2064/2354 (87.7)	2510/2889 (86.9)
CHART diagnosis at 3 y			
High risk	178/2511 (7.1)	166/2354 (7.0)	NA
Moderate risk	597/2511 (23.8)	564/2354 (24.0)	NA
Low risk	1736/2511 (69.1)	1624/2354 (69.0)	NA
Positive mAPI at 3 y	57/2480 (2.3)	55/2325 (2.4)	64/3371 (1.9)

Abbreviations: CHART, CHILDhood Asthma Risk Tool; ED, emergency department; mAPI, modified Asthma Predictive Index; NA, not applicable.

^a^ Data are presented as number/total number (percentage) of events unless otherwise indicated.

^b^ Other race or ethnicity includes First Nations, Hispanic, Metis, Middle Eastern, and participants who selected other (eg, Trinidadian and Brazilian).

^c^ Between 2 and 3 years of age.

### Prevalence of Wheeze, Asthma, and Health Care Burden at 3 Years

At 3 years of age, 414 of the 2511 children (16.5%) reported wheeze, of whom 220 (53.1%) had 2 or more episodes. Among those 220 children, 171 (77.7%) reported use of asthma medication at the same age. Overall, 250 of 2511 children (10.0%) were assigned an asthma diagnosis at 3 years of age by 1 or more definitions. Overall, of the 2511 children included at 3 years of age, 178 (7.1%) were classified as being at high risk of asthma, 597 (23.8%) at moderate risk, and 1736 (69.1%) at low risk (eFigure 1 in the Supplement). In-study specialist pediatricians deemed 141 children (5.6%) to have definite asthma and 169 (6.7%) to have possible asthma, whereas 112 (4.5%) had a parental report of external physician–diagnosed asthma. The mAPI was positive in only 57 (2.3%) of the 2480 children with available skin prick test data at 3 years (Table). Of the 2511 children, 247 of 2509 (9.8%) had used bronchodilators, and 277 of 2509 (11.0%) had used corticosteroids at least once; 81 of 2321 children (3.5%) required ED visits or hospitalizations for asthma or wheezing (Table). At 3 years of age, CHART identified 57 children at high risk for asthma not identified by physicians (eTable 3 in the Supplement).

### Prediction of Persistent Wheeze, Asthma, and Health Care Burden at 5 Years

**Figure 2.**
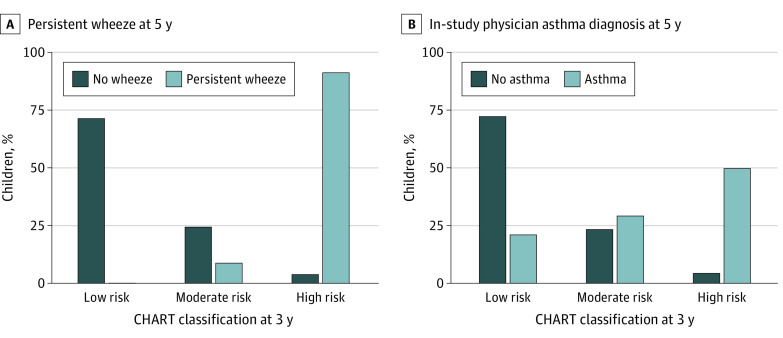
True-Positive and True-Negative Asthma and Persistent Wheeze at 5 Years by CHILDhood Asthma Risk Tool (CHART)

**Figure 3.**
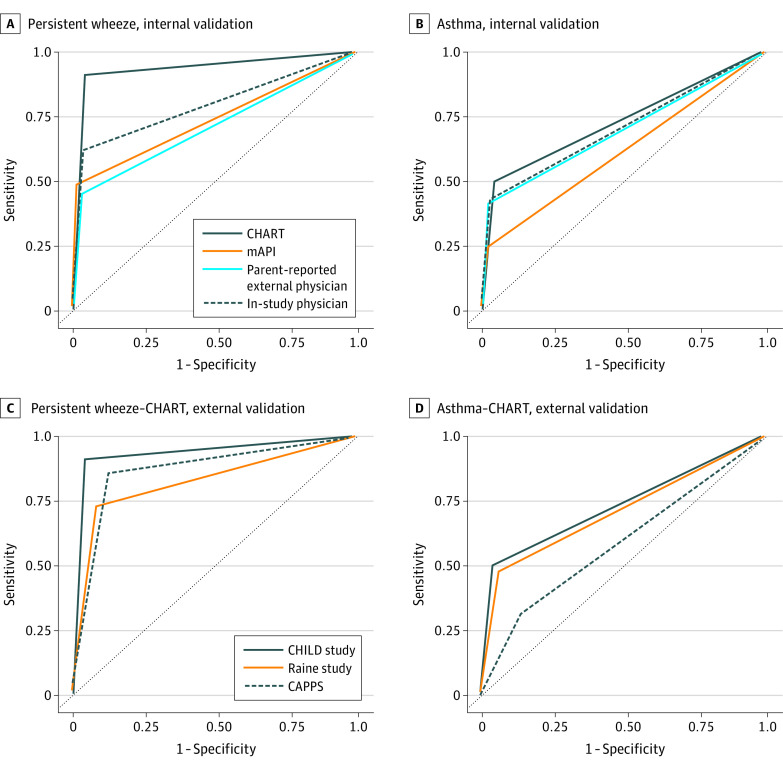
Area Under the Receiver Operating Characteristic Curves A and B, Prediction of persistent wheeze and asthma diagnosis at 5 years by alternative methods of asthma diagnosis at 3 years in the CHILD Study. C and D, CHILDhood Asthma Risk Tool (CHART) prediction of school-age persistent wheeze and asthma in the Raine Study and Canadian Asthma Primary Prevention Study cohorts compared with the CHILD Study (CAPPS). mAPI indicates modified Asthma Predictive Index.

Of the 220 children with 2 or more wheeze episodes at 3 years of age, 79 (35.9%) continued to experience wheeze to 5 years of age. CHART was more successful in identifying these persistent wheezers than physician diagnoses (eFigure 3 in the Supplement), classifying 72 of those 79 children as high risk (sensitivity, 91.1%; AUROC, 0.94; 95% CI, 0.90-0.97) (Figure 2A), whereas in-study physician diagnosis at 3 years identified only 49 (sensitivity, 62.0%; AUROC, 0.79; 95% CI, 0.74-0.85), and mAPI only 33 (sensitivity, 48.5%; AUROC, 0.74; 95% CI, 0.68-0.80) (Figure 3A; eTable 4 in the Supplement). In contrast, only 5 children were not identified by CHART but identified by physician diagnosis (n = 4) or mAPI (n = 1) (eFigure 4 in the Supplement). Specificity and NPV were consistent among all methods, whereas PPV was highest in mAPI (60.0%) followed by CHART (43.4%).

Our screening tool, based on symptom report up to 3 years of age, had the highest proportion of true-positive asthma at 5 years (sensitivity, 50.0%; AUROC, 0.73; 95% CI, 0.69-0.77) (Figure 2B), followed by the in-study physician diagnosis at 3 years (sensitivity, 43.5%; AUROC, 0.77; 95% CI, 0.73-0.81), parental report of external physician–diagnosed asthma ever (sensitivity, 41.7%; AUROC, 0.70; 95% CI, 0.65-0.74), and finally by positive mAPI at 3 years (sensitivity, 24.4%; AUROC, 0.62; 95% CI, 0.58-0.65) (Figure 3B; eTable 4 in the Supplement). Specificity and NPV were similar in all methods, whereas the highest PPV was achieved by mAPI (56.4%).

Among 367 children reporting current asthma medication use or ED visits or hospitalizations at 3 years of age (current supporting criteria) (eTable 2 in the Supplement), only 178 (48.5%) were classified as being at high risk by CHART, whereas the rest were recommended to have a follow-up assessment within 6 or 12 months. Of importance, CHART provided the highest predictive capacity for subsequent health care use at 5 years of age, identifying 20% more children with ED visits or hospitalizations (AUROC, 0.70; 95% CI, 0.61-0.78) than the standardized mAPI (sensitivity, 45.5% vs 25.0%) and approximately 10% more than the in-study physician (sensitivity, 36.4%) and external physician diagnosis (34.4%) (eTable 5 in the Supplement). Although CHART also had the highest sensitivity and AUROC for corticosteroid and bronchodilator use, mAPI had slightly higher specificity and PPV. Performance of Pediatric Asthma Risk Score to predict asthma at 5 years of age was similar to that obtained by CHART.

To evaluate the added benefit of predictors obtained from invasive tests, a sensitivity analysis restricted to the 2325 children (92.6%) with skin prick test data available at 3 years was performed. CHART continued to have higher sensitivity and AUROC than alternative methods of asthma diagnoses, including mAPI (eTable 6 in the Supplement). Furthermore, inclusion of blood eosinophilic levels of 1527 children at 1 year of age as an additional predictor did not improve the performance of CHART for diagnosis of asthma (sensitivity, 47.2%; AUROC, 0.71; 95% CI, 0.66-0.76) or persistent wheeze (sensitivity, 95.6%; AUROC, 0.95; 95% CI, 0.92-0.99).

### Prediction of Outcomes in External Cohorts

In the Raine Study, 2185 children had available data to apply CHART at 3 years of age. Of 301 children (13.8%) diagnosed with asthma at 5 years of age, 145 (48.2%) were identified as being at high risk by CHART at 3 years. Wheeze data were available for 1943 children, of whom 129 (6.6%) reported wheeze at 3 years that persisted to 5 years; 94 (72.9%) of those were identified by CHART at 3 years. The discriminative ability of CHART was similar to that obtained in CHILD for prediction of persistent wheeze (sensitivity, 72.9%; AUROC, 0.82; 95% CI, 0.79-0.86) and asthma (sensitivity, 48.2%; AUROC, 0.71; 95% CI, 0.68, 0.74) but higher for prediction of medication use (inhaled corticosteroid use: sensitivity, 60.8%; AUROC, 0.76; 95% CI, 0.72-0.80; bronchodilator or oral corticosteroid use: sensitivity, 58.7%; AUROC, 0.74; 95% CI, 0.69-0.79) at 5 years (Figure 3C-D; eTables 4-5 in the Supplement).

In the high-risk CAPPS cohort, 349 children had sufficient data to apply CHART at 2 years of age and predict outcomes at 7 years of age. CHART had a high discriminative capacity for identifying children with wheeze persisting to 7 years of age; among 28 children with persistent wheeze, 24 were classified as being at high risk at 2 years (sensitivity, 85.7%; AUROC, 0.87; 95% CI, 0.80-0.94). From 58 children diagnosed with asthma at 7 years of age, 19 were classified as being at high risk at 2 years of age by CHART (sensitivity, 32.8%; AUROC, 0.59; 95% CI, 0.52-0.65) (Figure 3C-D; eTable 5 in the Supplement). Predicted ability for medication use at 7 years of age was similar to that obtained in CHILD (eTable 5 in the Supplement). Notably, only 12 children reported ED visits for wheeze between the ages of 6 and 7 years, of whom 4 were categorized by CHART as being at high risk of asthma.

## Discussion

In this diagnostic study, we developed a simple screening tool capable of identifying 3-year-old children from the general population at risk of long-term burden from persistent symptoms of wheeze, asthma, and health care use. To allow broad applicability, CHART follows the standardized format shown in Figure 1, targeting information that can be easily gathered by health care practitioners through interviews and parent-reported questionnaires in primary care or low-resource settings. CHART easily flags children at high risk for further investigation by their primary care clinicians, whereas moderate-risk and low-risk children can be monitored regularly (preferably after 6 and 12 months) through incorporation into electronic medical records. It is important to note that CHART is designed as a pragmatic screening tool to help busy primary care clinicians identify the small proportion of children at high risk for persistent wheezing (7% in our population) among all children who report wheeze (42% at any time point). Once children are identified as being at high risk, clinicians will need to evaluate this smaller group for both severity and endotype of asthma. To our knowledge, this is the first study to develop a noninvasive tool for early detection of asthma and persistent wheeze in a general population that has subsequently been validated in general and high-risk cohorts. Next steps for high-risk individuals include the study of biomarkers for different endotypes of preschool asthma, which is necessary to improve our precision-based approach to asthma therapy.

As demonstrated in the external CAPPS cohort, CHART can be implemented at as early as 2 years of age; at 3 years, it outperformed physician assessments and the mAPI in detecting children who would have asthma at 5 years of age and in predicting persistent burden of symptoms and health care use. The sensitivity of CHART for asthma and persistent wheeze was consistently higher than standard diagnostic methods, highlighting its potential as a screening tool, maximizing true-positive results with only marginally decreased specificity compared with other diagnosis, such as mAPI. Although CHART does not differentiate asthma endotypes, it identified a smaller heterogeneous group of children at high risk of persistent symptoms within the general population who could be targeted for further evaluation. Increasing research points to the potential use of biomarkers, such as blood eosinophils, to help guide therapeutic choices, such as inhaled corticosteroids vs antileukotrienes in this age group.^23^ Although biological traits obtained from invasive methods are vital for identification of these asthma endotypes and targeted treatments, CHART allows the appropriate rationalization of these invasive and more expensive tests to a smaller group of high-risk children in whom these tests are more helpful to direct treatment.

Besides the Asthma Predictive Index and mAPI, other tools have been developed to detect asthma in children early in life, such as the Pediatric Asthma Risk Score and the Persistent Asthma Predictive Score.^13^ Unfortunately, these tools have the drawback of requiring tests (eg, spirometry, allergy skin tests, and blood tests) that limit their utility and implementation.^6,8,9,10,14,15^ In this study, we show that the inclusion of eosinophilic measures and skin prick tests did not improve performance of CHART; rather, they reduced our sample size by 40%, reflecting the difficulties with instituting invasive screening tests in the general population. Moreover, CHART performed similarly to the Pediatric Asthma Risk Score (the only other tool validated in a general population^13^) without requiring skin prick tests or incorporating race into its scale.^16^ Prediction tools that involve demographic characteristics, such as race, are often developed by regression modeling techniques. These techniques tend to incorporate variables specific to the population in which they are developed,^13,16,24^ potentially hindering their performance in low-resource or ethnically diverse settings.

In the CHILD Study, symptom-related items, such as wheezing in the past year, were closely related to an asthma diagnosis. Of note, CHART had the highest sensitivity for prediction of persistent wheeze (eFigure 4 in the Supplement), which has been deemed the most important symptom for identification of asthma.^25,26^ We considered a series of combinations in which not only the presence of wheeze but also the number of episodes and their co-occurrence with other conditions inform possible persistence. The strength of this approach is shown by the external validation in other cohorts. Our unique approach to classification of wheeze symptoms is important because it helps busy practitioners identify the smaller subset of children with more frequent or severe wheezing episodes who have a higher probability of continued symptoms and impaired lung function in adult life^27^ among most children with infrequent wheeze. Further work is needed to evaluate the types of asthma or endotypes identified using CHART.

Clinician diagnosis of asthma in preschool children is challenging because of the burden of symptoms as well as the variability of symptom severity and remission. However, the annual rate of ED visits and hospitalizations for asthma or wheeze in preschool children is twice that of individuals aged 6 to 70 years.^27^ Each hospitalization of Canadian children is associated with approximately 8 ED visits.^4^ We demonstrate that, in the CHILD, Raine, and CAPPS cohorts, CHART is as valuable in predicting burden due to ED visits and hospitalizations as the current criterion standard of physician asthma diagnoses. These findings are especially important given that many hospitalizations are avoidable if appropriate treatment and management of asthma are implemented at primary care.^4^

The implications of our results extend to epidemiologic and clinical domains. The finding that a symptom-based diagnosis of asthma performed better than multiple other methods of asthma diagnosis challenges the most common operational definition of diagnosis in epidemiologic studies, a parental report of physician-diagnosed asthma.^28^ Dichotomized definitions of asthma in epidemiologic studies, coupled with reluctance to label younger children as having asthma, may oversimplify the heterogeneity of asthma and hinder detection of young children with active disease.

Implementation of standardized tools to distinguish children at risk of persistent asthma symptoms and health care burden should trigger more active monitoring and in-depth assessment of physiology and biomarkers necessary for targeted phenotyping, thereby guiding therapy.^23^ Although early identification of asthma in children may not change the natural history of the disease, a current goal of asthma treatment is to minimize symptom burden and risk of asthma attacks.^2,29^ The implementation of CHART as a first-step screening tool in general practice could promote timely treatment control and, in turn, improve quality of life for patients and reduce the clinical and economic burden of asthma.^30^

### Limitations

Although a strength of our study is that it was undertaken in a multiethnic cohort of children, we recognize that CHART was developed and validated in children mostly living in urban centers. A future direction could be testing CHART in low-resource and remote settings to confirm its predictive performance. This study also had some additional limitations. The Raine Study and CAPPS did not collect all measures required at preschool age to calculate mAPI (eg, skin prick tests and allergic rhinitis diagnosis); therefore, only comparisons between clinical diagnosis of asthma and CHART were possible in those cohorts. In addition, the sensitivity (50%) and PPV (41.5%) for asthma in CHART are suboptimal for a screening test. The striking contrast to the high performance obtained for persistent wheeze suggests that this may be rooted in physicians’ hesitance to diagnose asthma at a young age. Monitoring and validation of CHART at primary care settings are needed to evaluate its utility in practice, and all predictors were gathered from parental questionnaires or clinical assessments, increasing the possibility of recall bias. To minimize this issue, CHART input was limited to symptom information. A potential solution to further reduce recall bias, if implemented into electronic medical records, could be to administer our 4-question screening tool to parents biannually (episodes of wheeze and cough in the past 6 months, ED visits and/or hospitalizations for wheeze or asthma attacks, and medication use).

## Conclusions

CHART is a validated tool that, in this diagnostic study, was able to identify children from 2 years of age at higher risk for persistent wheeze and more likely to develop asthma in general and high-risk populations. The tool could potentially be implemented routinely as part of electronic medical records initiated at infancy as a simple, noninvasive screening tool for children at primary care.

## Notes

Supplement. **eMethods.** Supplemental Methods **eAppendix.** Supplemental Results
eReferences
**eTable 1. **Supporting Criteria for CHART Diagnosis at Age 3 in the CHILD Cohort Study **eTable 2.** Inclusion Criteria and Outcome Definitions in CHILD, CAPPS and *R*aine *C*ohorts **eTable 3.** Agreement, Sensitivity, Specificity, Positive Predictive Value and Negative Predictive Value Among Operational Definitions of Asthma at Age 3 Years **eTable 4.** Predictive Accuracy of Alternative Definitions of Asthma Diagnoses Persistent Wheeze at 2-3 Years, Asthma in the CHILD Cohort Study (5 Years), Raine Study (5 Years) and CAPPS (7 Years) **eTable 5.** Predictive Performance of CHART and Alternative Asthma Diagnosis at 3 Years for Medication and Health Care Utilization **eTable 6.** Sensitivity Analysis: Alternative Definitions for Asthma Diagnoses at 3 Years and Outcomes at 5 Years Restricted to Children With Available Skin Prick Test Data in CHILD (N=2325) **eFigure 1.** CHILD Asthma Risk Tool (CHART) Classification in 2511 Children at Age 3 Years in the CHILD Cohort Study **eFigure 2.** CHILD Cohort Study Consort Diagram Indicating Participants Included in Study Sample **eFigure 3.** Persistent Wheeze Detection **eFigure 4.** Venn Diagram of Wheeze Persisting to Age 5 in CHILD
