# Supplementary material for: Development of a Symptom-Based Tool for Screening of Children at High Risk of Preschool Asthma
Source: JAMA Netw Open. 2022 Oct 6;5(10):e2234714. doi: 10.1001/jamanetworkopen.2022.34714 (PMC9539714; doi:10.1001/jamanetworkopen.2022.34714)
Supplement: Supplement. — eMethods. Supplemental Methods eAppendix. Supplemental Results eReferences eTable 1. Supporting Criteria for CHART Diagnosis at Age 3 in the CHILD Cohort Study eTable 2. Inclusion Criteria and Outcome Definitions in CHILD, CAPPS and Raine Cohorts eTable 3. Agreement, Sensitivity, Specificity, Positive Predictive Value and Negative Predictive Value Among Operational Definitions of Asthma at Age 3 Years eTable 4. Predictive Accuracy of Alternative Definitions of Asthma Diagnoses Persistent Wheeze at 2-3 Years, Asthma in the CHILD Cohort Study (5 Years), Raine Study (5 Years) and CAPPS (7 Years) eTable 5. Predictive Performance of CHART and Alternative Asthma Diagnosis at 3 Years for Medication and Health Care Utilization eTable 6. Sensitivity Analysis: Alternative Definitions for Asthma Diagnoses at 3 Years and Outcomes at 5 Years Restricted to Children With Available Skin Prick Test Data in CHILD (N=2325) eFigure 1. CHILD Asthma Risk Tool (CHART) Classification in 2511 Children at Age 3 Years in the CHILD Cohort Study eFigure 2. CHILD Cohort Study Consort Diagram Indicating Participants Included in Study Sample eFigure 3. Persistent Wheeze Detection eFigure 4. Venn Diagram of Wheeze Persisting to Age 5 in CHILD [file jamanetwopen-e2234714-s001.pdf]

Supplementary Online Content

Reyna ME, Dai R, Tran MM, et al. Development of a symptom-based tool for screening of children at high risk of preschool asthma. *JAMA Netw Open*. 2022;5(10):e2234714. doi:10.1001/jamanetworkopen.2022.34714

eMethods. Supplemental Methods
eAppendix. Supplemental Results
eReferences
eTable 1. Supporting Criteria for CHART Diagnosis at Age 3 in the CHILD Cohort Study
eTable 2. Inclusion Criteria and Outcome Definitions in CHILD, CAPPS and Raine Cohorts
eTable 3. Agreement, Sensitivity, Specificity, Positive Predictive Value and Negative Predictive Value Among Operational Definitions of Asthma at Age 3 Years
eTable 4. Predictive Accuracy of Alternative Definitions of Asthma Diagnoses and Persistent Wheeze at 2-3 Years, Asthma in the CHILD Cohort Study (5 Years), Raine Study (5 Years) and CAPPS (7 Years)
eTable 5. Predictive Performance of CHART and Alternative Asthma Diagnosis at 3 Years for Medication and Health Care Utilization
eTable 6. Sensitivity Analysis: Alternative Definitions for Asthma Diagnoses at 3 Years and Outcomes at 5 Years Restricted to Children With Available Skin Prick Test Data in CHILD (N=2325)
eFigure 1. CHILD Asthma Risk Tool (CHART) Classification in 2511 Children at Age 3 Years in the CHILD Cohort Study
eFigure 2. CHILD Cohort Study Consort Diagram Indicating Participants Included in Study Sample
eFigure 3. Persistent Wheeze Detection
eFigure 4. Venn Diagram of Wheeze Persisting to Age 5 in CHILD

This supplementary material has been provided by the authors to give readers additional information about their work.

© 2022 Reyna ME et al. *JAMA Network Open.*

eMethods. Supplemental Methods

Study Design

The CHILD cohort study recruited 3,621 Canadian pregnant women resulting in 3455 children who met eligibility criteria at birth [1]. Parent reported questionnaires on child health and clinical assessments were completed at multiple time points up to 5 years. Parents answered questions drawn from the International Study of Asthma and Allergies in Childhood (ISAAC) [2], and children were examined for asthma and other allergic diseases. For this study, children with sufficient data at age, 3, and 5 years were included in the analysis (eFigure 1).

This study was approved centrally by the Hamilton Integrated Research Ethics Board (HiREB #07-2929) and local ethics boards.

Inclusion and exclusion criteria in the CHILD cohort study

Inclusion Criteria:

[1] pregnant women aged 18 years and older (19 in Vancouver);

[2] residence in reasonable proximity to the delivery hospital;

[3] able to read, write, and speak English;

[4] willing to provide informed consent;

[5] willing to consent to cord blood collection for the study;

[6] planning to give birth at a designated recruitment centre participating hospital;

[7] infants born at or after 35 weeks gestation;

[8] able to provide name, address and telephone numbers of two alternate contact individuals.

Exclusion Criteria:

[1] children born with major congenital abnormalities or respiratory distress syndrome (RDS);

© 2022 Reyna ME et al. *JAMA Network Open.*

[2] expectation of moving away from a recruitment area within 1 year;

[3] children of multiple births;

[4] children resulting from in vitro fertilization with external manipulation of the ova;

[5] children who will not spend at least 80% of nights in the index home;

[6] children born before 35 weeks gestation.

Final enrolment was confirmed postpartum.

Alternative methods for asthma diagnosis

a) **In-study physician diagnosis (current gold standard):** An in-study pediatric asthma specialist conducted a structured interview with the accompanying parent or guardian identifying symptoms consistent with asthma, and noted any physical findings. Criteria used to diagnose asthma may include, but were not limited to, standardized clinical histories, objective evidence and symptoms gathered during in person assessments when the child was 3 and 5 years of age [1]. The physician then answered the question: "In your opinion, does this child have asthma? (Yes / Possible / No)". Children in the "possible" category were considered as "No asthma".

b) **Parent-reported external physician diagnosis**: At age 3 years, a questionnaire asked parents, "Has your child ever been diagnosed with asthma? (Yes / No / Don't Know)". Positive responses were recorded as 'parent-reported external physician asthma'. All other responses, including no record of visits to an external physician for asthma-related symptoms, were recorded as a negative response.

c) **Modified Asthma Predictive Index (mAPI):** A positive mAPI requires ≥4 wheezing episodes during the past year, together with one of three major criteria (physician-diagnosed eczema, parental asthma, or allergic sensitization to ≥1 aeroallergen) or two of

© 2022 Reyna ME et al. *JAMA Network Open.*

three minor criteria (wheezing without colds, physician-diagnosed allergic rhinitis or allergic sensitization to milk, egg, or peanuts) [3].

Current "Asthma" Diagnosis at 3 Years

CHART classification at 3 years was compared to in-study physician diagnosis, parental report of physician-diagnosed asthma ever, and the mAPI at 3 years (Table E3). Agreement between all asthma definitions of cases and non-cases was compared by Cohen's kappa coefficient (95% confidence interval).

Supporting Criteria for screening tool.

Children who had wheezed or who had had ≥2 episodes of cough were further categorized according to number of wheeze episodes. Those with ≥2 episodes of wheeze in the last 12 months (current wheeze) and 1 or more supporting criteria were categorized as having "Definite asthma". Supporting criteria included an emergency department (ED) visit for asthma/wheezing, hospitalization for asthma/wheezing, inhaled or oral corticosteroid (CS) use, bronchodilator (BD) use, or frequent dry cough at night, during exercise, or when laughing or crying.

Health Care Utilization

Health care utilization in the CHILD cohort study was analyzed by parent-reported questionnaire data at the time of the 5-year study visit.

Bronchodilator and inhaled corticosteroid use was defined as a positive answer to any of the questions "Was your child given any bronchodilators (asthma relievers) in the last year?" and "Was your child given any treatment(s) with corticosteroid inhalers or nebule(s) during the last year?", or if the use of specific drugs were reported in medication questionnaires.

© 2022 Reyna ME et al. *JAMA Network Open.*

ED visits and hospitalizations were deemed positive if parents responded yes to the question: "Has your child had any ER or Urgent Care visits/ hospital admissions/stays in the last year?" and the subsequent question "What was the reason(s) for these ER or Urgent Care visit(s)?" with the optional answers "wheezing episode" and/or "asthma attack".

Skin Prick Tests at 3 and 5 years

Skin prick tests at 3 and 5 years to standardized inhalant allergens and common food allergens (ALK Abello Pharmaceuticals Canada) were applied to children by trained staff using Duotip II devices (Lincoln Diagnostics Canada). Food allergens included: peanut, milk, egg white and soy; while inhalant allergens included: alternaria tenuis, cat hair, dog epithelium, house dust mites (Der.p and Der.f), cockroach, penicillium, aspergillus fumigatus, cladosporium, trees, grasses, weeds and ragweed. Individual wheal sizes for allergens or positive (histamine 0.1%) and negative (glycerin) controls were determined by the mean of the longest diameter and its perpendicular measured at the mid-point of the maximum length, both recorded in millimeters to one decimal place. The mean size of the negative control wheal, if present, was subtracted from the mean size of each individual allergen response. Allergen responses ≥2mm were accepted as positive even if the histamine wheal was <2mm, but children were excluded from analysis if there was no histamine response and no allergen response.

External validation cohorts

The Raine Study

The Western Australian Pregnancy Cohort (Raine) study is a longitudinal birth cohort that recruited 2868 Australian pregnant women born between 1989 and 1992. The study gathered data related to asthma outcomes at multiple time points throughout childhood, from which we

© 2022 Reyna ME et al. *JAMA Network Open.*

extracted information on children's symptoms at ages 3 and 5 years to define inclusion criteria and perform validation of CHART. Details on the Raine study population have been published elsewhere [4]. Children of enrolled families were labelled as asthmatic at age 5 in the presence of all of the following: parental report of physician diagnosis of asthma, wheezing or cough in the absence of a cold over the last 12 months, and taking asthma medication in the preceding 12 months by parental report [4].

For this study, a total of 2185 Raine participants had data available to apply CHART at 3 years and data on asthma at age 5, among whom 1943 had available data for the outcome of persistent wheeze to age 5. Skin prick tests at age 3 years and ED visits at age 5 years were not available in the Raine study.

The CAPPS cohort

The Canadian Asthma Primary Prevention Study (CAPPS) is a prospective, prenatally randomized, controlled clinical trial of 545 Canadian children at high-risk of asthma development [5]. Enrolled children were born between 1994 and 1996 to at least one first-degree relative with asthma or 2 first-degree relatives with other classic IgE-mediated allergic diseases. Data from parental questionnaires and physician interviews were used to define inclusion criteria, and children were assessed for possible and probable asthma at 2 and 7 years of age. Details on clinical assessments in CAPPS can be found elsewhere [6,7]. Possible asthma was defined as at least 2 distinct episodes of cough, each lasting for 2 or more weeks; at least 2 distinct episodes of wheeze, each lasting 2 or more weeks; or in the absence of a cold, at least one of the following: nocturnal cough (at least once a week) and hyperpnea-induced cough or wheeze (eg, with crying, laughing, or activity). Probable asthma was defined as at least 2 distinct episodes of cough, each lasting 2 or more weeks, or at least 2 distinct episodes of wheeze, each

© 2022 Reyna ME et al. *JAMA Network Open.*

lasting 1 or more weeks, and at least one of the following: nocturnal cough (at least once a week) in the absence of cold, hyperpnea-induced cough or wheeze, response to treatment with β-agonist, anti-inflammatory drugs, or both. For this study, asthma at age 7 was defined as having physician diagnosed asthma plus bronchial hyper responsiveness (PC_{20} < 4 mg/mL) [7]. Interventions in this cohort took place prior to collection of symptom data for CHART, thus, both intervention (n=189) and control (n=160) groups were included in the analysis. A sensitivity analysis restricted to participants in the control group showed that predictive ability of CHART did not change substantially for asthma (AUROC=0.56, sensitivity=30%) nor for wheeze persisting to age 7 (AUROC=0.80, sensitivity=75%). Allergic rhinitis data at 2 years and hospitalization data at 7 years were not available in the CAPPS cohort.

© 2022 Reyna ME et al. *JAMA Network Open.*

eAppendix. Supplemental Results

CHART compared to Pediatric Asthma Risk Cohort (PARS)

Diagnostic accuracy of our screening tool was compared to that obtained from the validated PARS, which requires data on parental asthma, history of eczema before 3 years, wheezing apart from colds, early wheezing (before 3 years), SPT to ≥2 aero and/or food allergens, and identification of African American participants [8]. In an initial analysis, CHILD participants were classified with eczema based on parent reported history of rash. African American ethnicity was identified when at least one parent self-identified as black. Under these definitions, CHART performed similarly to PARS, with an AUROC of 0.73 (95% CI 0.69, 0.77), sensitivity=0.65, and specificity=0.80. However, AUROC and sensitivity of PARS decreased using a more conservative approach that consisted on defining eczema as in-study clinician diagnosis, and assigning African American race only when both parents self-identified as black (AUROC= 0.69 (95%CI 0.65, 0.74), sensitivity=0.48, specificity=0.89).

Sensitivity analysis on children with skin prick tests in the CHILD Cohort Study

Results from a sensitivity analysis on the predictive ability of CHART were consistent to the findings from the main analysis (Table 3). In children with available skin prick tests, 5.5% had in-study physician diagnosed asthma at 5 years and 2.9% had wheeze that persisted to age 5. Our screening tool applied at age 3 years was more successful in predicting persistence of wheeze to 5 years than physician diagnoses (Sensitivity =91%, AUROC 0.94), compared to in-study physician diagnosis (Sensitivity=62%, AUROC=0.79), and mAPI (Sensitivity=49%, AUROC=0.74).

© 2022 Reyna ME et al. *JAMA Network Open.*

eReferences

1. Subbarao P, Anand SS, Becker AB, et al. The Canadian Healthy Infant Longitudinal Development (CHILD) Study: examining developmental origins of allergy and asthma. *Thorax*. 2015;70(10):998-1000. doi:10.1136/thoraxjnl-2015-207246

2. Worldwide variations in the prevalence of asthma symptoms: the International Study of Asthma and Allergies in Childhood (ISAAC). *Eur Respir J*. 1998;12(2):315-335.

3. Guilbert TW, Morgan WJ, Zeiger RS, et al. Atopic characteristics of children with recurrent wheezing at high risk for the development of childhood asthma. *J Allergy Clin Immunol*. 2004;114(6):1282-1287. doi:10.1016/j.jaci.2004.09.020

4. Joseph-Bowen J, De Klerk NH, Firth MJ, Kendall GE, Holt PG, Sly PD. Lung function, bronchial responsiveness, and asthma in a community cohort of 6-year-old children. *American Journal of Respiratory and Critical Care Medicine*. 2004;169(7):850-854. doi:10.1164/rccm.200304-556oc

5. Chan-Yeung M, Manfreda J, Dimich-Ward H, Ferguson A, Watson W, Becker A. A randomized controlled study on the effectiveness of a multifaceted intervention program in the primary prevention of asthma in high-risk infants. *Archives of Pediatrics and Adolescent Medicine*. 2000;154(7):657-663. doi:10.1001/archpedi.154.7.657

6. Becker A, Watson W, Ferguson A, Dimich-Ward H, Chan-Yeung M. The Canadian asthma primary prevention study: Outcomes at 2 years of age. *Journal of Allergy and Clinical Immunology*. 2004;113(4):650-656. doi:10.1016/j.jaci.2004.01.754

7. Chan-Yeung M, Ferguson A, Watson W, et al. The Canadian Childhood Asthma Primary Prevention Study: Outcomes at 7 years of age. *Journal of Allergy and Clinical Immunology*. 2005;116(1):49-55. doi:10.1016/j.jaci.2005.03.029

8. Biagini Myers JM, Schauberger E, He H, et al. A Pediatric Asthma Risk Score to better predict asthma development in young children. *Journal of Allergy and Clinical Immunology*. 2019;143(5):1803-1810. doi:10.1016/j.jaci.2018.09.037

© 2022 Reyna ME et al. *JAMA Network Open.*

eTable 1. Supporting Criteria for CHART Diagnosis at Age 3 in the CHILD Cohort Study. (eFigure 1)

	**≥2 episodes of wheeze ever n=510**		**≥2 cough episodes and no wheeze ever n= 334**		**1 wheeze episode and ≥2 cough episodes n=132**		**1 wheeze episode and/or 1 cough episode n=268**	
**Current symptoms (last 12 months)**	Current n=220	Not current n=290	Current n=160	Not current n=174	Current n=39	Not current n=93	Current n=66	Not current n=202
**Supporting criteria**								
ER visit for wheeze or asthma	59	9	0	0	6	0	8	0
Hospitalization for wheeze or asthma	8	0	0	0	0	0	1	0
Use of inhaled/oral corticosteroids	124	43	21	5	11	6	15	7
Use of inhaled bronchodilator	144	38	20	4	8	6	8	3
Frequent cough*	95	38	130	0	22	26	0	0
**One or more of the above, N (%)**	**178 (80.9%)**	**75 (26.2%)**	**135 (84.4%)**	**6 (3.4%)**	**30 (76.9%)**	**31 (33.3%)**	**24 (36.4%)**	**8 (4.0%)**

*Frequent cough defined as ≥2 episodes of cough without a cold **and** cough at night, during exercise, or when laughing or crying at 3 years of age.

eTable 2. Inclusion Criteria and Outcome Definitions in CHILD, CAPPS and Raine Cohorts.

Variable	CHILD Inclusion criteria<br>(2.5 and 3 years)	CAPPS inclusion criteria<br>(1.5 and 2 years)	Raine inclusion criteria<br>(3 years)
**Wheeze Episodes**	*At 2.5 and 3 years:*<br>In the last 6 months, has your child had a wheezing noise (whistling sound) coming from his/her chest either WITH a cold or WITHOUT a cold?<br>- If Yes, how many episodes?	*At 1.5 years and 2 years:*<br>Did the baby have any wheezing episodes since the 1-year visit?<br>- If yes, how many episodes since the 1-year visit?<br>Has the baby had a wheeze at 2-year assessment?<br>- If Yes, how many episodes?	*At 3 years:*<br>Has your child's chest ever sounded wheezy or whistling in the past 12 months?<br>When he/she has a cold?<br>- If yes, how many times<br>Occasionally even without a cold?<br>- If yes, how many times<br>- Most days or nights? Yes/no<br>- After exercise/vigorous play? Yes/no
**Cough Episodes**	*At 2.5 and 3 years:*<br>Has your child had any coughing episodes WITHOUT a cold in the last 6 months?<br>- If Yes, how many episodes?	*At 1.5 years and 2 years:*<br>Has the baby had any cough since last visit?<br>Was the cough brought by a cold?<br>*At 2 years:*<br>Has the baby had a cough?<br>- If Yes, how many episodes?<br>Has the baby had a cough without apparent "colds"?<br>Does this cough happen during the night?<br>- If Yes, Hyperpnoea induced (laugh, cry)?<br>- If Yes, Exercise?<br>- If Yes, At rest?	*At 3 years:*<br>Does he/she cough frequently even without colds these days?<br>- Yes/no
**ED Visits**	*At 2.5 and 3 years:*<br>What was the reason(s) for these ER or Urgent Care visit(s)?<br>- Wheezing episode<br>- Asthma attack	*At 1.5 years and 2 years:*<br>Were the wheeze episodes severe (hospital visit)?	*At 3 years:*<br>Has your child had any illnesses or problems in the past 12 months which required you to take him/her to the hospital, doctor or clinic<br>- If yes, describe
**Hospital Admissions**	*At 2.5 and 3 years:*<br>What was the reason(s) for these hospital stay(s)?<br>- Wheezing episode<br>- Asthma attack	Not asked	*At 3 years:*<br>Has your child needed to be admitted to any hospital in the past 12 months?<br>- If yes, which hospitals and what for (Asthma or wheeze)

© 2022 Reyna ME et al. *JAMA Network Open.*

**Broncho-dilator**	*At 2.5 and 3 years:*<br>Was your child given any bronchodilators (asthma relievers) in the last 6 months?	*At 1.5 years and 2 years:*<br>Was Oral/Inhaled Beta Agonist given for these coughing or wheezy episodes?<br>*At 2 years:*<br>Oral β-Agonist?<br>MDI β-Agonist?<br>Wet nebulized β-Agonist?	*At 3 years:*<br>Is your child taking any prescription medicine now, either regularly or on occasion?<br>- Bronchodilators<br>- Oral steroids<br>- Inhaled steroids<br>- Cromoglycate<br>- Other medications
**Cortico-steroids**	*At 2.5 and 3 years:*<br>Was your child given any treatment(s) with corticosteroid inhalers or nebule(s) in the last 6 months?	*At 1.5 years and 2 years:*<br>Was Inhaled Steroid given for these coughing or wheezy episodes?<br>Was Systemic Steroid Oral/IV/IM given for these coughing or wheezy episodes?<br>Inhaled Corticosteroids MDI at 2 year assessment?<br>Inhaled Corticosteroids Aerosolized?<br>Oral or IV corticosteroids?	
**Frequent Cough**	*At 2.5 and 3 years:*<br>Has your child had any coughing episodes WITHOUT a cold in the last 6 months<br>Does your child cough at night while asleep?<br>Does your child cough when crying?<br>Does your child cough when laughing?	*At 2 years:*<br>Has the baby had a cough without apparent "colds"?<br>Does this cough happen during the night?<br>- If Yes, Hyperpnoea induced (laugh, cry)?<br>- If Yes, Exercise?<br>- If Yes, At rest?	*At 3 years:*<br>Does he/she cough most days or nights, either all year round or at least part of the year?<br>Has he/she had an attack of coughing, congestion or bringing up phlegm lasting for more than 1 week in the past 12 months?
**Outcome**	**Outcomes in CHILD (5 years)**	**Outcomes in CAPPS (7 years)**	**Outcomes in Raine (5 years)**
**Asthma**	*In-study physician diagnosis:*<br>In your opinion, does the child have any of the following? Asthma:<br>- Yes/possible/no<br>*Parental report of external physician diagnosis:*<br>Has your child ever been diagnosed with asthma?	Possible or probable asthma diagnosed by in-study physician and the presence of bronchial hyper responsiveness ($PC_{20} < 4$)	Presence of parental report of physician diagnosis of asthma, plus wheezing or a cough in the absence of a cold over the last 12 months, plus taking asthma medication in the preceding 12 months by parental report.
**Persistent Wheeze**	2 or more episodes of wheeze with or without colds at 3 and 5 years	2 or more episodes of wheeze with or without colds at 2 and 7 years	2 or more episodes of wheeze with or without colds at 3 and 5 years

© 2022 Reyna ME et al. *JAMA Network Open.*

eTable 3. Agreement, Sensitivity, Specificity, Positive Predictive Value and Negative Predictive Value Among Operational Definitions of Asthma at Age 3 Years.
Values calculated using alternating 'standard' (the second definition in each row).

Test/ 'standard'	Agreement among cases	Agreement among non-cases	Kappa (95% CI)	Sensitivity (%)	Specificity (%)	PPV (%)	NPV (%)
CHART/ In-study Physician	100/141 (70.9%)	2292/2370 (96.7%)	0.60 (0.53, 0.67)	70.9	96.7	56.2	98.2
In-study Physician / CHART	100/178 (56.2%)	2292/2333 (98.2%)		56.2	98.2	70.9	96.7
CHART/ External Physician	68/112 (60.7%)	2289/2399 (95.4%)	0.44 (0.35, 0.52)	60.7	95.4	38.2	98.1
External Physician/ CHART	68/178 (38.2%)	2289/2333 (98.1%)		38.2	98.1	60.7	95.4
External Physician/ In-study Physician	60/141 (42.6%)	2318/2370 (97.8%)	0.45 (0.36, 0.54)	42.6	97.8	53.6	96.6
In-study Physician / External Physician	60/112 (53.6%)	2318/2399 (96.6%)		53.6	96.6	42.6	97.8
CHART/ mAPI	53/57 (93.0%)	2322/2423 (95.8%)	0.49 (0.39, 0.58)	93.0	95.8	34.4	99.8
mAPI/ CHART	53/154 (34.4%)	2322/2326 (99.8%)		34.4	99.8	93.0	95.8
In-study Physician / mAPI	36/57 (63.2%)	2335/2423 (96.4%)	0.38 (0.26, 0.49)	63.2	96.4	29.0	99.1
mAPI/ In-study Physician	36/124 (29.0%)	2335/2356 (99.1%)		29.0	99.1	63.2	96.4
External Physician/ mAPI	29/57 (50.9%)	2347/2423 (96.9%)	0.34 (0.21, 0.46)	50.9	96.9	27.6	98.8
mAPI/ External Physician	29/105 (27.6%)	2347/2375 (98.8%)		27.6	98.8	50.9	96.9

Pairwise association tests between each of the 3 methods of asthma diagnoses and mAPI all have p-values < 0.0001**.**

© 2022 Reyna ME et al. *JAMA Network Open.*

eTable 4. Predictive Accuracy of Alternative Definitions of Asthma Diagnoses at 2-3 Years and Persistent Wheeze, Asthma in the CHILD Cohort Study (5 years), Raine Study (5 years) and CAPPS (7 years)

Persistent wheeze								
**2-3 years diagnosis**		**No (%)**	**Yes (%)**	**AUROC (95%CI)**	**PPV (95%CI)**	**NPV (95%CI)**	**Sensitivity (95%CI)**	**Specificity (95%CI)**
**CHILD (Age 5)**								
CHART*	Low	1459 (64.1)	0 (0.0)	0.94 (0.90, 0.97)	43.4 (35.7, 51.3)	99.7 (99.3, 99.9)	91.1 (82.6, 96.4)	95.9 (95, 96.6)
	Moderate	722 (31.8)	7 (8.9)					
	High	94 (4.1)	72 (91.1)					
Parent-report	No	2101 (96.7)	40 (54.8)	0.71 (0.65, 0.77)	31.7 (22.9, 41.6)	98.1 (97.5, 98.7)	45.2 (33.5, 57.3)	96.7 (95.9, 97.4)
	Yes	71 (3.3)	33 (45.2)					
In-study physician	No†	2192 (96.4)	30 (38.0)	0.79 (0.74, 0.85)	37.1 (28.9, 46)	98.6 (98.1, 99.1)	62.0 (50.4, 72.7)	96.4 (95.5, 97.1)
	Yes	83 (3.6)	49 (62.0)					
mAPI	Negative	2235 (99.0)	35 (51.5)	0.74 (0.68, 0.80)	60.0 (45.9, 73.0)	98.5 (97.9, 98.9)	48.5 (36.2, 61.0)	99.0 (98.5, 99.4)
	Positive	22 (1.0)	33 (48.5)					
**Raine (Age 5)**								
CHART*	Low/Moderate	1668 (92.0)	35 (27.1)	0.82 (0.79, 0.86)	39.2 (33, 45.7)	97.9 (97.2, 98.6)	72.9 (64.3, 80.3)	92.0 (90.6, 93.2)
	High	146 (8.0)	94 (72.9)					
**CAPPS (Age 7)**								
CHART*	Low	0 (0.0)	0 (0.0)	0.87 (0.80, 0.94)	38.1 (26.1, 51.2)	98.6 (96.5, 99.6)	85.7 (67.3, 96)	87.9 (83.8, 91.2)
	Moderate	284 (87.9)	4 (14.3)					
	High	39 (12.1)	24 (85.7)					
**Asthma**								
**2-3 years diagnosis**		**No (%)**	**Yes (%)**	**AUROC (95%CI)**	**PPV (95%CI)**	**NPV (95%CI)**	**Sensitivity (95%CI)**	**Specificity (95%CI)**
**CHILD (Age 5)**								
CHART*	Low	1433 (64.7)	26 (18.8)	0.73 (0.69, 0.77)	41.5 (34.0, 49.5)	96.8 (96.0, 97.5)	50.0 (41.4, 58.6)	95.6 (94.7, 96.4)
	Moderate	686 (31.0)	43 (31.2)					
	High	97 (4.4)	69 (50.0)					
Parent-report	No	2064 (97.7)	77 (58.3)	0.70 (0.65, 0.74)	52.9 (42.8, 62.8)	96.4 (95.5, 97.2)	41.7 (33.2, 50.6)	97.7 (96.9, 98.3)
	Yes	49 (2.3)	55 (41.7)					
In-study physician	No†	2144 (96.7)	78 (56.5)	0.77 (0.73, 0.81)	45.5 (36.8, 54.3)	96.5 (95.6, 97.2)	43.5 (35.1, 52.2)	96.7 (95.9, 97.4)
	Yes	72 (3.2)	60 (43.5)					
mAPI	Negative	2174 (98.9)	96 (75.6)	0.62 (0.58, 0.65)	56.4 (42.3, 69.7)	95.8 (94.9, 96.6)	24.4 (17.2, 32.8)	98.9 (98.4, 99.3)
	Positive	24 (1.1)	31 (24.4)					
**Raine (Age 5)**								
CHART*	Low/Moderate	1759 (93.4)	156 (51.8)	0.71 (0.68, 0.74)	53.7 (47.6, 59.8)	91.9 (90.5, 93)	48.2 (42.4, 54)	93.4 (92.1, 94.4)
	High	125 (6.6)	145 (48.2)					
**CAPPS (Age 7)**								
CHART*	Low	0 (0.0)	0 (0.0)	0.59 (0.52, 0.65)	30.2 (19.2, 43)	86.4 (81.8, 90.1)	32.8 (21, 46.3)	84.9 (80.2, 88.8)
	Moderate	249 (84.9)	39 (67.2)					
	High	44 (15.1)	19 (32.8)					

*Low and moderate risk groups classified as "No risk" for calculation of AUROC, PPV, NPV, Sensitivity and Specificity.

†Includes a physician diagnosis of no asthma and possible asthma.

© 2022 Reyna ME et al. *JAMA Network Open.*

eTable 5. Predictive Performance of CHART and Alternative Asthma Diagnosis at 3 Years for Medication and Health Care Utilization.

Calculated using data from the CHILD Cohort Study (5 years), Raine Study (5 years) and CAPPS (7 years).

2-3 years diagnosis	Health care utilization	AUROC (95%CI)	PPV (95%CI)	NPV (95%CI)	Sensitivity (95%CI)	Specificity (95%CI)
**CHILD (Age 5)**						
CHART	Inhaled Corticosteroid use	0.64 (0.61, 0.67)	52.2 (44.2, 60.1)	91.4 (90.1, 92.5)	31.3 (25.8, 37.3)	96.2 (95.3, 97)
	Oral Corticosteroid use	0.58 (0.54, 0.61)	18.0 (12.4, 24.8)	94.9 (93.9, 95.8)	21.2 (14.7, 29.0)	93.8 (92.8, 94.8)
	Bronchodilator use	0.66 (0.63, 0.68)	62.7 (54.8, 70.2)	90.9 (89.6, 92.1)	34.1 (28.7, 39.8)	97.0 (96.2, 97.7)
	ED visits/ hospitalization	0.70 (0.61, 0.78)	11.1 (6.4, 17.7)	99.1 0.99, 0.99)	45.5 (28.0, 64.0)	94.1 (93.0, 95.0)
mAPI	Inhaled Corticosteroid use	0.56 (0.54, 0.59)	66.0 (51.7, 78.5)	90.1 (88.8, 91.3)	13.8 (9.8, 18.6)	99.1 (98.6, 99.5)
	Oral Corticosteroid use	0.55 (0.52, 0.57)	28.3 (16.8, 42.3)	94.5 (93.5, 95.5)	11.1 (6.4, 17.7)	98.2 (97.5, 98.7)
	Bronchodilator use	0.57 (0.55, 0.59)	75.5 (61.7, 86.2)	89.2 (87.8, 90.4)	14.3 (10.4, 18.9)	99.3 (98.9, 99.7)
	ED visits/ hospitalization	0.62 (0.54, 0.69)	18.6 (8.4, 33.4)	98.8 (98.2, 99.2)	25.0 (11.5, 43.4)	98.3 (97.6, 98.8)
In-study physician diagnosis	Inhaled Corticosteroid use	0.63 (0.60, 0.66)	58.9 (49.9, 67.5)	91.1 (89.9, 92.3)	28.4 (23, 34.2)	97.4 (96.6, 98.0)
	Oral Corticosteroid use	0.56 (0.53, 0.60)	18.7 (12.4, 26.6)	94.8 (93.7, 95.7)	17.5 (11.6, 24.9)	95.2 (94.2, 96.0)
	Bronchodilator use	0.63 (0.60, 0.66)	65.1 (56.2, 73.3)	90.2 (88.9, 91.4)	28.4 (23.3, 33.9)	97.8 (97.0, 98.4)
	ED visits/ hospitalization	0.66 (0.57, 0.74)	11.1 (5.9, 18.6)	98.9 (98.4, 99.3)	36.4 (20.4, 54.9)	95.3 (94.2, 96.1)
Parent-reported external physician diagnosis	Inhaled Corticosteroid use	0.61 (0.59, 0.64)	62.4 (52.2, 71.8)	90.9 (89.6, 92.1)	24.9 (19.7, 30.7)	98.0 (97.3, 98.6)
	Oral Corticosteroid use	0.56 (0.53, 0.59)	20.6 (13.2, 29.7)	94.6 (93.5, 95.5)	15.7 (10, 23)	96.0 (95.1, 96.8)
	Bronchodilator use	0.62 (0.60, 0.65)	72.3 (62.5, 80.7)	90.3 (88.9, 91.5)	26.4 (21.3, 32.1)	98.5 (97.9, 99)
	ED visits/hospitalization	0.65 (0.57, 0.74)	12.8 (6.6, 21.7)	98.9 (98.3, 99.3)	34.4 (18.6, 53.2)	96.1 (95.2, 96.9)
**Raine (Age 5)**						
CHART	Inhaled Corticosteroid use	0.76 (0.72, 0.80)	32.4 (26.5, 38.6)	97.1 (96.1, 97.8)	60.8 (51.8, 69.2)	91.1 (89.7, 92.3)
	Bronchodilator/Oral CS use	0.74 (0.69, 0.79)	22.1 (17.1, 27.9)	97.8 (97, 98.4)	58.7 (47.9, 68.9)	89.9 (88.5, 91.2)
	Hospitalizations	0.79 (0.72, 0.85)	15.6 (11.3, 20.7)	99.0 (98.4, 99.4)	67.9 (54, 79.7)	89.3 (87.8, 90.6)
**CAPPS (Age 7)**						
CHART	Inhaled Corticosteroid use	0.58 (0.50, 0.65)	31.2 (18.7, 46.3)	83.7 (77.6, 88.6)	32.6 (19.5, 48)	82.8 (76.7, 87.9)
	Oral corticosteroid use	0.57 (0.46, 0.69)	12.8 (4.8, 25.7)	93.8 (89.3, 96.7)	33.3 (13.3, 59)	81.4 (75.7, 86.3)
	Bronchodilator use	0.61 (0.55, 0.67)	44.4 (31.9, 57.5)	81.8 (76.9, 86.1)	35.0 (24.7, 46.5)	87.0 (82.4, 90.8)
	ED visits	0.58 (0.44, 0.72)	6.4 (1.8, 15.5)	97.2 (94.6, 98.8)	33.3 (9.9, 65.1)	82.5 (78.0, 86.4)

© 2022 Reyna ME et al. *JAMA Network Open.*

eTable 6. Sensitivity Analysis: Alternative Definitions for Asthma Diagnoses at 3 Years and Outcomes at 5 Years Restricted to Children With Available Skin Prick Test Data in CHILD (n=2325).

Persistent wheeze to age 5					
**3 years diagnosis**	**AUROC (95%CI)**	**PPV**	**NPV**	**Sensitivity**	**Specificity**
CHART	0.94 (0.90, 0.97)	43.4 (35.1, 51.9)	99.7 (99.4, 99.9)	91.2 (81.8, 96.7)	96.4 (95.6, 97.1)
Parent-report	0.73 (0.66, 0.79)	30.9 (21.9, 41.1)	98.5 (97.9, 99.0)	48.4 (35.5, 61.4)	96.9 (96.1, 97.6)
In-study physician	0.79 (0.73, 0.85)	36.5 (27.7, 46.0)	98.8 (98.3, 99.2)	61.8 (49.2, 73.3)	96.8 (96.0, 97.5)
mAPI	0.74 (0.68, 0.80)	60.0 (45.9, 73.0)	98.5 (97.9, 98.9)	48.5 (36.2, 61.0)	99.0 (98.5, 99.4)
**Asthma at age 5**					
**3 years diagnosis**	**AUROC**	**PPV**	**NPV**	**Sensitivity**	**Specificity**
CHART	0.71 (0.67, 0.75)	40.6 (32.4, 49.1)	96.8 (96.0, 97.5)	45.7 (36.8, 54.7)	96.1 (95.2, 96.9)
Parent-report	0.70 (0.65, 0.74)	52.6 (42.2, 62.8)	96.7 (95.8, 97.4)	41.8 (32.9, 51.1)	97.8 (97.1, 98.4)
In-study physician	0.69 (0.65, 0.73)	45.2 (35.9, 54.8)	96.6 (95.8, 97.3)	40.9 (32.3, 50.0)	97.1 (96.3, 97.8)
mAPI	0.62 (0.58, 0.65)	56.4 (42.3, 69.7)	95.8 (94.9, 96.6)	24.4 (17.2, 32.8)	98.9 (98.4, 99.3)

© 2022 Reyna ME et al. *JAMA Network Open.*

Figures

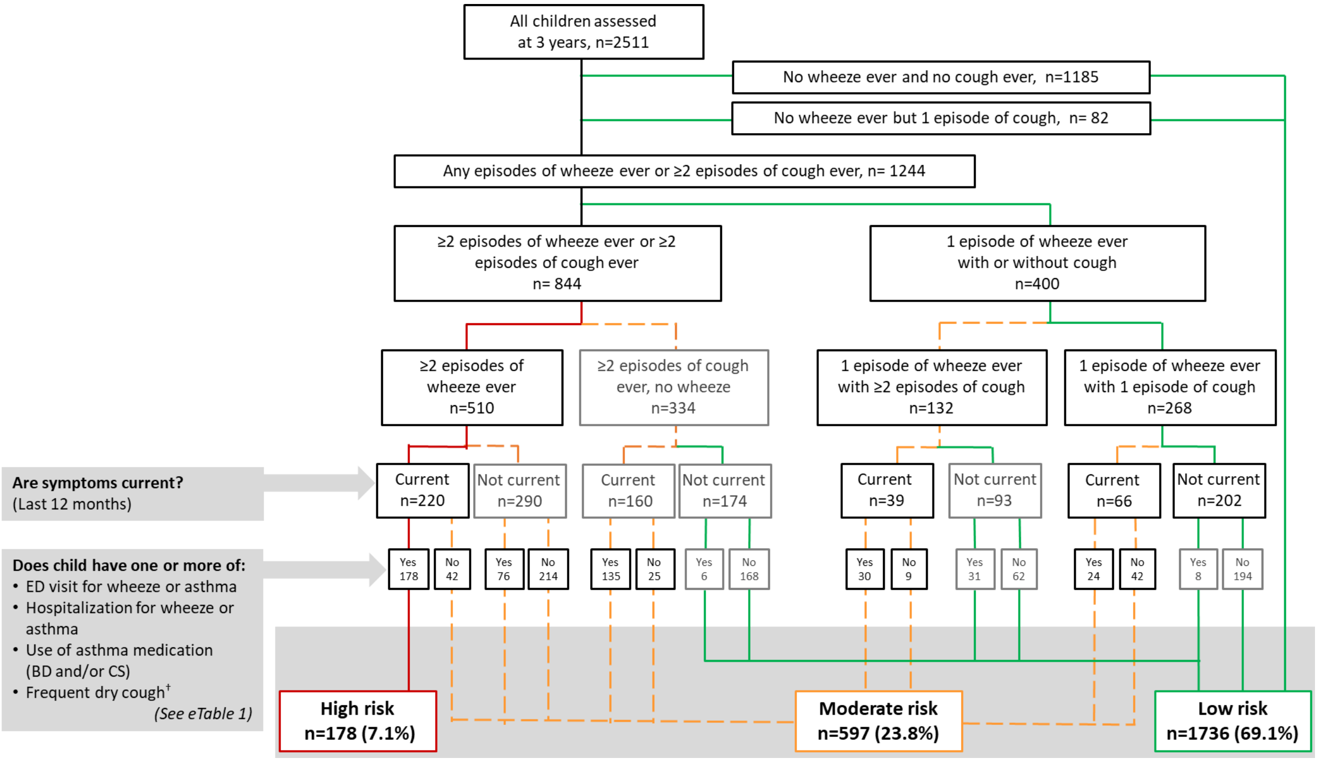

eFigure 1. CHILD Asthma Risk Tool (CHART) Classification in 2511 Children at Age 3 Years in the CHILD Cohort Study.

Based on parent-reported child symptoms, healthcare utilization, and medication use. Details of supporting data presented in Table E1.

†Includes cough at night

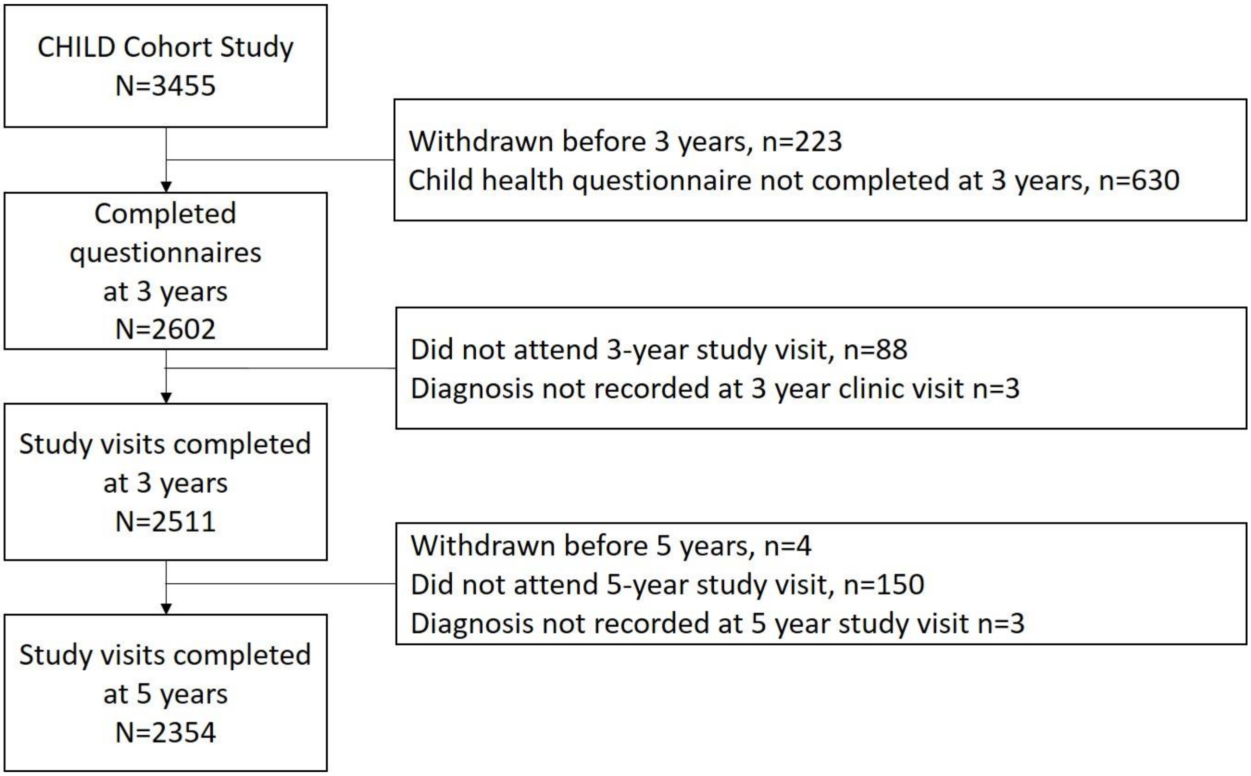

eFigure 2. CHILD Cohort Study Consort Diagram Indicating Participants Included in Study Sample.

© 2022 Reyna ME et al. *JAMA Network Open.*

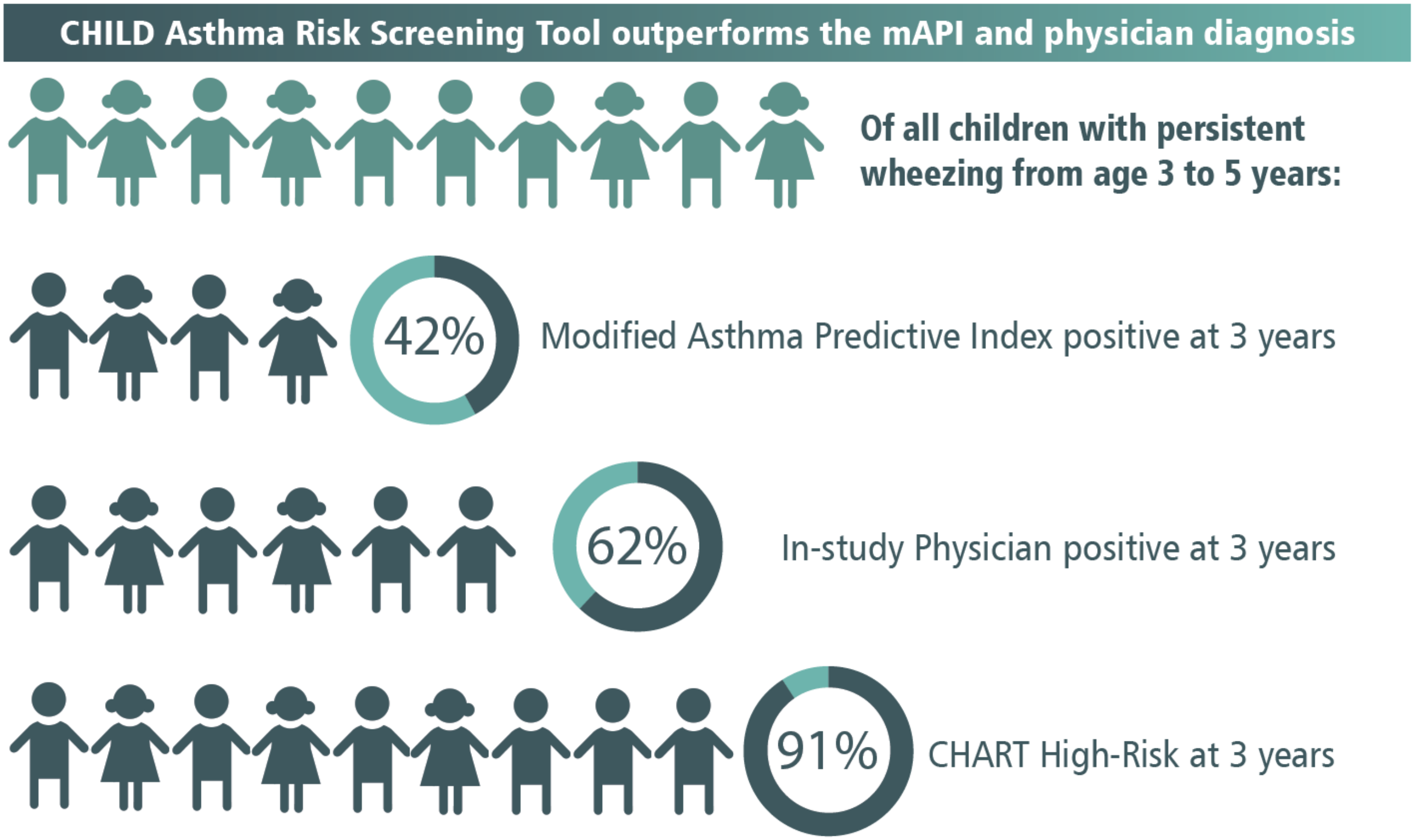

eFigure 3. Persistent Wheeze Detection

© 2022 Reyna ME et al. *JAMA Network Open.*

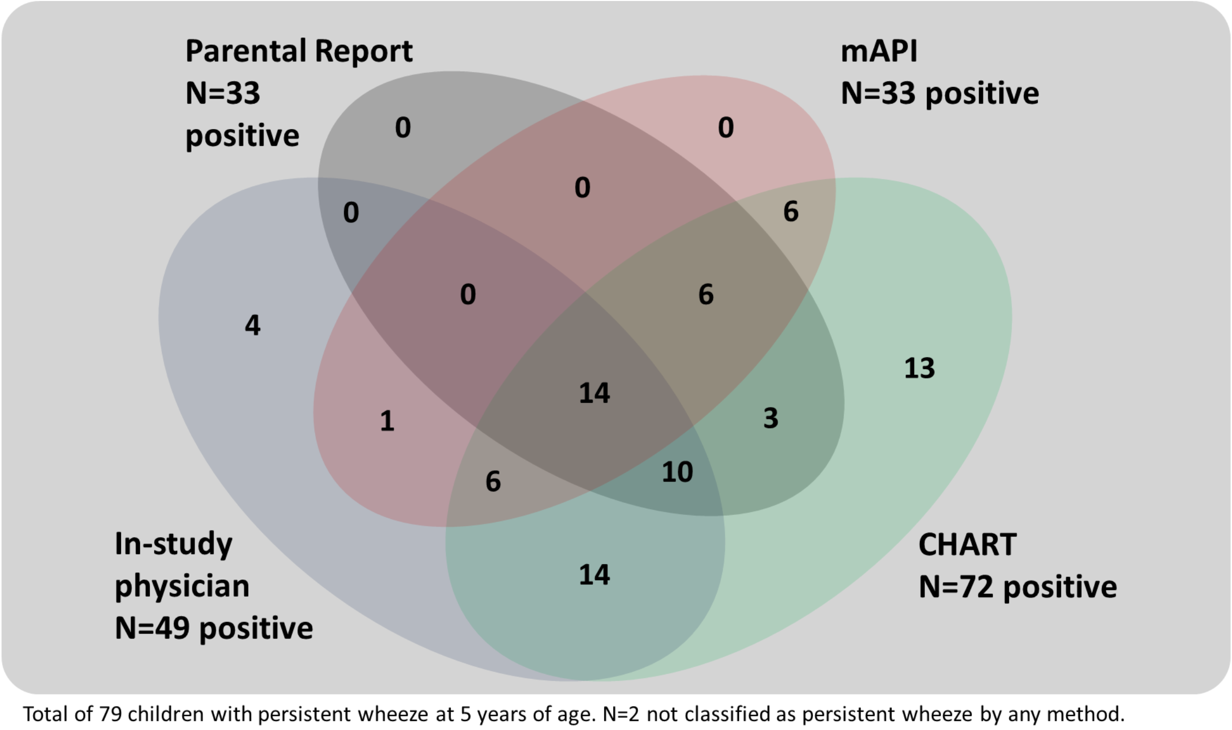

eFigure 4. Venn Diagram of Wheeze Persisting to Age 5 in CHILD.
Persistent wheeze cases (N=79) by three years' asthma diagnosis by CHART, in-study physician diagnosis, parent-reported external physician diagnosis and modified API.

© 2022 Reyna ME et al. *JAMA Network Open.*
